# Effect of stocking density on growth performance, gross energy, proximate analysis of meat, and stress response of mahseer (*Tor soro*) fry

**DOI:** 10.7717/peerj.21034

**Published:** 2026-04-01

**Authors:** Tri Heru Prihadi, Brata Pantjara, Deni Radona, Titin Kurniasih, Amran Ronny Syam, Adang Saputra, Armen Zulham, Ena Sutisna, Muhamad Yamin, Yuani Mundayana, Lili Sholichah, Sri Turni Hartati, Irin Iriana Kusmini, Agus Oman Sudrajat, Djamhuriyah S. Said, Safar Dody, Tutik Kadarini, Estu Nugroho, Andi Parenrengi

**Affiliations:** 1 Research Center for Freshwater Aquaculture, National Research and Innovation Agency, Bogor, West Java, Indonesia; 2 Research Center for Applied Zoology, National Research and Innovation Agency, Bogor, West Java, Indonesia; 3 Research Center for Biota System, National Research and Innovation Agency, Bogor, West Java, Indonesia; 4 Research Center for Cooperative, Corporation, and People’s Economy, National Research and Innovation Agency, Jakarta, Indonesia; 5 Department of Aquaculture, IPB University, Bogor, West Java, Indonesia; 6 Research Center for Limnology and Water Resources, National Research and Innovation Agency, Bogor, West Java, Indonesia; 7 Research Center for Oceanography, National Research and Innovation Agency, Serpong, South Tanggerang, Indonesia

**Keywords:** Growth, Physiology, Stocking density, *Tor soro*

## Abstract

Mahseer fish (genus *Tor*) is a freshwater species with significant cultural and economic value in Southeast Asia, but its development remains hindered by limitations in aquaculture technology. The study aims to evaluate the effect of different stocking densities on growth performance, feed efficiency, survival rates, energy retention, stress response, and proximate analysis of meat in juvenile *Tor soro*. The experiment was conducted in a completely randomized design with varying stocking density levels, namely one fish/L, two fish/L, three fish/L, and four fish/L as treatment, with four replicates each. Findings indicate that different stocking densities had a significant effect on the aforementioned growth-related parameter where the stocking density of one fish/L showed the highest weight gain (4.36 ± 0.42 grams), the fastest daily growth rate (1.17 ± 0.09% per day), the highest gross energy value (2,151 kcal/g), the most efficient feed conversion ratio or FCR (1.76 ± 0.13), the lowest cortisol levels (6.06 ± 0.19 ng/ml), and the lowest superoxide dismutase (SOD) levels (3.50 ± 0.89 ng/mg), compared to the other treatments. These results indicate that a stocking density of one individual/L was the optimal density for mahseer seed rearing in culture containers.

## Introduction

Mahseers (genus *Tor*) are ecologically and economically important freshwater fish distributed across the trans-Himalayan and Southeast Asian river systems (Abass et al., 2024; Jaafar et al., 2021). Of the 50 globally recognized species, Indonesia hosts four: *Tor soro*, *T. tambroides*, *T. tambra*, and *T. douronensis* (Muchlisin et al., 2022). Wild *T. soro* populations, however, continue to decline due to overexploitation, habitat degradation, pollution, and hydrological disruptions affecting spawning and nursery areas (Akmal et al., 2022; Setijaningsih et al., 2023). Despite these pressures, *T. soro* remains valued for its rapid growth, aquaculture adaptability, and high flesh quality (Chasanah et al., 2021). Increasing demand for consumption and sport fishing necessitates sustainable aquaculture expansion, yet large-scale production is hindered by suboptimal stocking densities and immature rearing technologies (Arifin et al., 2020). Biologically, *T. soro* is an omnivore feeding on algae, plants, insects, and crustaceans, with juveniles relying more on invertebrates (Everard et al., 2021; Indra et al., 2024). It is an active swimmer inhabiting clean, fast-flowing rivers and exhibiting seasonal feeding and monsoon-driven reproductive patterns (Arnenda et al., 2023; Dwivedi, 2021; Rai et al., 2025).

Stocking density is a critical determinant of aquaculture efficiency. Elevated densities increase competition, disease transmission, waste accumulation, and water quality deterioration, cumulatively impairing growth, immunity, and physiological stability (El Nouman et al., 2021; Gullian-Klanian & Arámburu-Adame, 2013; Sundh et al., 2019). Density-related effects are widely reported across species which include *Anguilla marmorata* (Tan et al., 2018), *Pelteobagrus fulvidraco* (Diao et al., 2023), *Micropterus salmoides* (Jia et al., 2022), and *Ompok bimaculatus* (Majhi et al., 2023). Thus, balancing stocking intensity with environmental management is essential (Abd El-Hack et al., 2022).

Previous trials with *T. soro* indicate substantial density-dependent variation in growth performance. Larvae stocked at ∼5,000 ind./m^3^ showed very low growth despite high survival (Subagja et al., 2021), while juveniles reared at 125 ind./m^2^ exhibited slow growth and poor condition (Arnenda et al., 2023), and RAS systems with ∼100 ind./m^3^ produced survival rates of 53.5–99%, influenced by system stability and density-related environmental pressure (Fadir et al., 2022). Similar density effects have been reported for *T. putitora* (Riar, Raushon & Paul, 2021). However, these findings remain fragmented because the studies differ widely in life stage, rearing system, and density range, making the results incomparable and preventing the establishment of practical stocking guidelines for *T. soro*. Furthermore, no previous study has assessed density-associated physiological stress responses or water-quality dynamics, despite the species’ sensitivity to environmental fluctuations. This gap is critical, as the lack of stage-specific density recommendations continues to hinder consistent seed production and the development of scalable aquaculture protocols. To address these gaps, the present study investigates the effects of defined stocking density gradients (1–4 fish/L) on growth performance, feed efficiency, survival, energy retention, body composition, stress biomarkers (cortisol, superoxide dismutase (SOD)), and water-quality stability in rearing juvenile *T. soro*, providing comprehensive data needed to establish biologically sound and operationally feasible density standards.

## Materials & Methods

The experiment was conducted at the Fish Hatchery and Raising Laboratory, Germplasm Installation, Research Institute for Freshwater Aquaculture and Fisheries Extension (RIFA-FE), Bogor, Indonesia, from December 2023 to April 2024. Water quality assessments were carried out at the Environmental Toxicology Laboratory and Aquaculture Environment Laboratory of RIFA-FE, and the Department of Aquaculture, Faculty of Fisheries and Marine Sciences, Institut Pertanian Bogor (IPB) University. Proximate composition and energy content analyses were performed at the Inter-University Central Laboratory and the Animal Nutrition Laboratory, Faculty of Animal Science, IPB University. Hormonal and enzymatic analyses (cortisol and SOD) were conducted at the Genetic Laboratory of RIFA-FE.

### Ethical statement

All experimental procedures were conducted in accordance with the Procedures and Guidelines for Animal Ethics of the Republic of Indonesia, authorized by the Ethics Commission for Animal Care and Use, National Research and Innovation Agency (BRIN), under approval Number: 105/KE.02/SK/05/2023.

### Experimental animals and design

This study used a completely randomized design (CRD) with four stocking density treatments: one fish/L, two fish/L, three fish/L, and four fish/L (*n* = 4 replicates per treatment). The study utilized *Tor soro* fry with an initial mean length of 5–6 cm and average weight of 2 g, for a total of 600 fish. The fry were obtained from artificial spawning at the Freshwater Fisheries Germplasm Research Installation, Cijeruk, Bogor. Sixteen glass aquaria (48  × 48  × 30 cm; effective volume 15 L) were randomly assigned to treatments. Fish were reared for 60 days under controlled environmental conditions with continuous aeration and daily water quality monitoring.

### Experimental setup and preparation

All aquaria were thoroughly scrubbed, disinfected using a chlorine solution, and sun-dried for three days before use. Each tank was equipped with a two× three cm air stone connected to a central aeration line. Based on prior observations identifying blue high-density paper as the optimal background color for reducing stress and improving performance, each aquarium was wrapped in blue HD paper. Dechlorinated freshwater was added to each aquarium and aerated for 48 h before stocking to stabilize water parameters. Baseline water conditions were adjusted and maintained at: 5 NTU turbidity, 7–8 mg/L dissolved oxygen, 75 mg/L alkalinity, and 6.5–7.5 pH, with values adjusted according to established protocols for *Tor soro* aquaculture. Aquaria were arranged randomly on identical racks within a temperature-controlled room to minimize environmental variation.

### Feed management and water quality control

Fish were fed a commercial pellet diet (PF500, PT Central Proteina Prima, Indonesia) containing approximately 32% crude protein and 5% crude lipid. Feeding was conducted three times daily (08:00, 13:00, and 17:00) at 5% of the fish biomass, with feed amounts adjusted every 10 days based on biomass sampling. However, the actual amount provided did not exactly match the pre-weighed 5%, as feeding was adjusted in accordance with the water-quality management protocols described below. The main factor affecting water quality deterioration in aquaculture systems is excessive feeding rather than infrequent water exchange. Therefore, feed management and routine waste removal were prioritized to maintain optimal environmental conditions. Feed was provided in quantities that could be fully consumed within 10–15 min, and the feeding rate was reduced by 10–20% when uneaten feed was consistently observed. In practice, the actual feed provided generally remained close to the targeted 5% ratio. The daily feed to be provided was always weighed and placed in plastic containers labeled for each experimental unit. Each afternoon, after the completion of feeding, the remaining feed in the plastic containers (*i.e.,* feed that was not given) was weighed again to determine the total amount of feed administered that day. The cumulative feed provided over the 60-day experimental period was also totaled for the calculation of the feed conversion ratio (FCR).

Accumulated feces and organic debris were removed daily *via* siphoning to prevent ammonia buildup and preserve water clarity. Additionally, a daily water exchange of approximately 30% was performed to maintain acceptable water quality, with the exchange rate adjusted according to biomass density, feeding intensity, and observed water quality trends. Strong aeration was provided continuously, 24 h a day, to ensure that oxygen levels remained at optimal levels. Water quality parameters (temperature, dissolved oxygen, turbidity, pH, nitrite, nitrate, alkalinity, total hardness, and total ammonia nitrogen (TAN)) were monitored daily to ensure stable experimental conditions. All measurements adhered to APHA (2017) standards.

### Growth performance and survival

Growth parameters were calculated from initial and final measurements (day 0 and day 60) using the following equations:

\begin{eqnarray*}\text{Body weight gain (g)}=\text{final weight (g)}-\text{initial weight (g)} \end{eqnarray*}

\begin{eqnarray*}\text{Daily growth rate}~(\%/\mathrm{day})= \frac{\text{ln [final weight (g)]}-\text{ln [initial weight (g)]}}{\text{Experimental period in days}} \end{eqnarray*}

\begin{eqnarray*}\text{Survival rate} \left( \% \right) = \frac{\text{Number of individuals after a period}}{\text{Number of individuals at the start}} \times 100. \end{eqnarray*}

### Proximate and gross energy analysis

Proximate composition was analyzed at both the start and end of the trial, whereas gross energy was measured only at the end of the feeding experiment. At the beginning, 30 fish from the stock population were analyzed. At the end, 10 fish per treatment were randomly sampled. Fish were euthanized using an overdose of MS-222 (200 mg/L, immersion for 1 min) and freeze-dried before analysis. Biochemical analyses of whole-body proximate components (moisture, crude protein, crude fat, and ash) of fish samples were conducted in accordance with the procedures outlined by Groot, Lyons & Schrama (2021). Dry matter (DM) was determined by drying the samples at 103 °C until a constant weight was reached (for 4 and 24 h, respectively). Ash content was measured after incineration at 550 °C for 4 h. Crude protein (CP) was calculated as nitrogen × 6.25 using the Kjeldahl method. Crude fat was analyzed following acid hydrolysis and subsequent extraction with petroleum-diethyl ether. Gross energy (GE) was measured using an adiabatic bomb calorimeter. Nitrogen-free extract (NFE) was determined as: DM – CP – Fat – Ash – Crude Fibre.

### Biochemical assays

Biochemical sampling was conducted at two time points: pre-treatment (day 0) and final sampling (day 60). Three fish from each treatment were anesthetized with MS-222 (75 mg/L) and euthanized with MS-222 (200 mg/L) for blood and liver tissue collection. Blood was drawn from the caudal vein using one mL heparinized syringes, while livers were excised immediately, weighed, and stored at −80 °C. Plasma cortisol was quantified using a competitive ELISA kit (Cortisol EIA Kit, Cayman Chemical, Ann Arbor, MI, USA). Absorbance was measured at 450 nm using a BioTek Synergy H1 microplate reader, and results expressed in ng/mL plasma. Hepatic superoxide dismutase SOD activity was determined following the epinephrine autoxidation method of Misra & Fridovich (1972) with slight modifications: Liver tissue (0.1 g) was homogenized in two mL phosphate buffer (pH 7.4), and then homogenates were centrifuged at 10,000 × g for 20 min at 4 °C. Furthermore, the supernatant was mixed with chloroform: ethanol (3:5, v/v), vortexed, and re-centrifuged (3,000 × g, 10 min). 100 µL of the resulting supernatant was added to three mL carbonate buffer (pH 10.2, 30 °C), followed by addition of epinephrine (0.05 mg/10 mL in 0.01 N HCl). Absorbance at 480 nm was then recorded at 1, 2, and 3 min using a spectrophotometer (UV-1800, Shimadzu, Japan). Controls included blank (0.01 N HCl) and sample-free control (distilled water). SOD activity was expressed as U mg^−^^1^ protein, where one unit represents the amount of enzyme causing 50% inhibition of epinephrine auto-oxidation.

### Statistical analysis

Survival rate, growth rate, feed conversion ratio (FCR), gross energy, proximate, and biochemical parameters (cortisol and SOD activity) were subjected to one-way analysis of variance (ANOVA) at a 95% confidence level (*α* = 0.05) to evaluate the effects of stocking density. Significant differences (*p* < 0.05) among treatment means were further analyzed using Duncan’s multiple range test (DMRT) for *post-hoc* comparisons. Water quality parameters were summarized using descriptive statistics (mean ± SD) and presented as temporal trends to assess environmental stability during the trial. All data were tested for normality (Shapiro–Wilk test) and homogeneity of variances prior to parametric analysis. Statistical computations were performed using Microsoft Excel (2019, Redmond, WA, USA) and Minitab 16.0 software. Results were reported as mean ± standard deviation (SD).

## Results

The growth performance parameters of juvenile mahseer (*Tor soro*) during the rearing period are presented in Table 1. All growth parameters in Mahseer fish were influenced by stocking density treatment (*P* < 0.05). The highest weight gain (4.36 ± 0.42 g), the highest length increase (6.89 ± 0.41 cm), the lowest weight gain (2.63 ± 0.05 g), the lowest length increase (5.57  ± 0.06 cm) was shown in the stocking density of four fish/L. The highest body length is in the density of one fish/L (1.60 ± 0.40 cm) and is significantly different compared to lower stocking densities. The lowest feed conversion ratio (FCR) was shown in the one fish/L treatment (1.76 ± 0.13) and significantly different compared to the stocking densities of two, three, and four fish/L.

**Table 1:** Growth performance of juvenile mahseer fish (*Tor soro*) with different stocking densities.

Parameters		Treatment		
	1 fish/L	2 fish/L	3 fish/L	4 fish/L
Initial body length (cm)	5.29 ± 0.04^b^	5.25 ± 0.04^ab^	5.26 ± 0.03^ab^	5.23 ± 0.02^a^
Initial body weight (gr)	2.15 ± 0.12^a^	2.20 ± 0.09^a^	2.14 ± 0.02^a^	2.15 ± 0.05^a^
Final body length (cm)	6.89 ± 0.41^c^	6.48 ± 0.19^b^	6.36 ± 0.03^b^	5.57 ± 0.06^a^
Final body weight (gr)	4.36 ± 0.42^c^	4.17 ± 0.06^bc^	3.83 ± 0.18^b^	2.63 ± 0.05^a^
Absolute length (cm)	1.60 ± 0.40^c^	1.23 ± 0.21^b^	1.10 ± 0.05^b^	0.34 ± 0.05^a^
Feed Conversion Ratio (FCR)	1.76 ± 0.13^a^	2.83 ± 0.14^b^	2.91 ± 0.20^b^	2.95 ± 0.13^b^

**Notes.**

Numbers followed by the same superscript letters in in the same row showed no significant difference (Duncan’s test *P* > 0.05). All values are expressed as mean ± SD.

**Figure 1:**
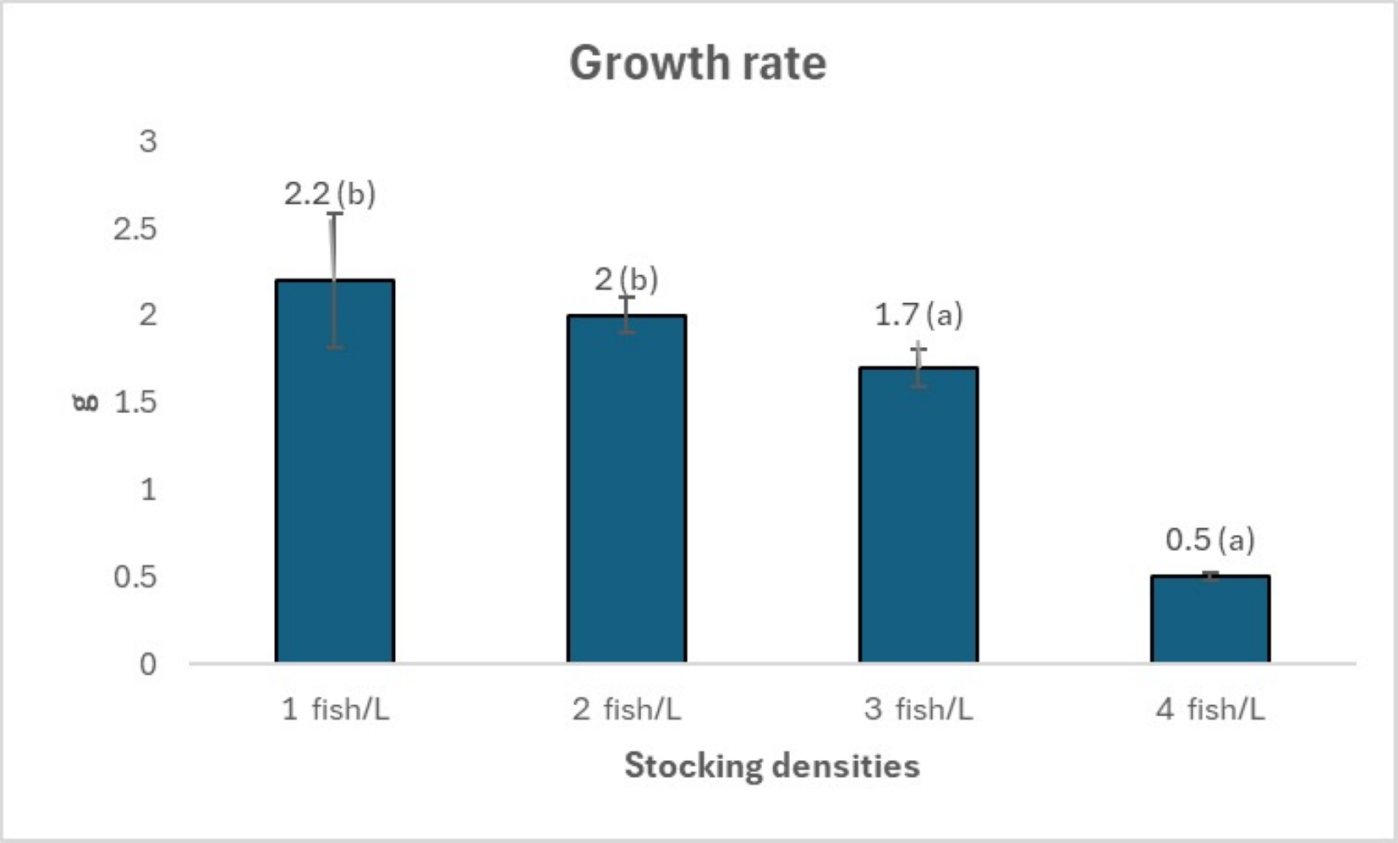
Growth rate of mahseer fish seeds in treatments with different stocking densities. Note: Numbers followed by the different superscript letters in the graph show significant difference (*P* < 0.05).

**Figure 2:**
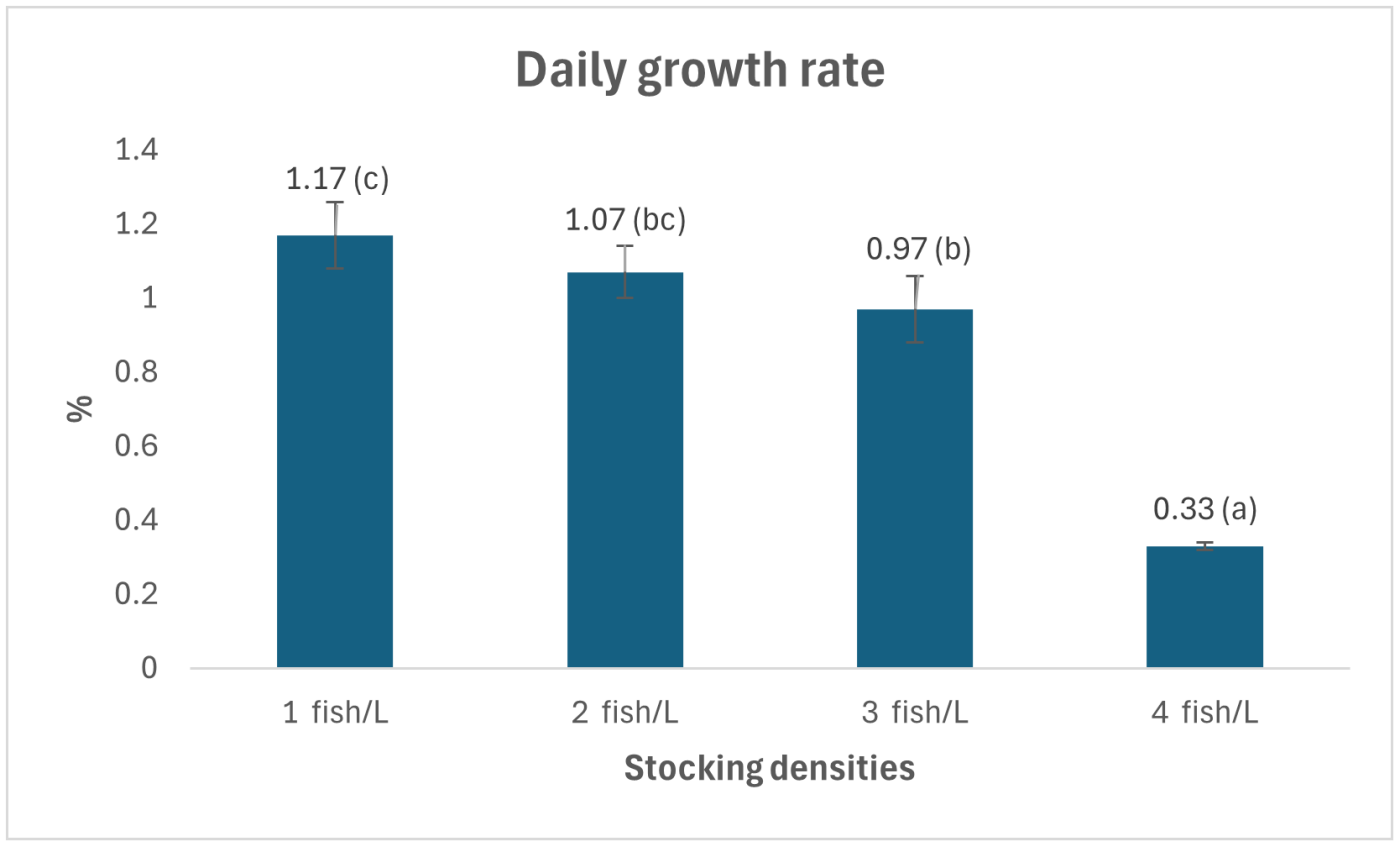
Daily growth rate of mahseer fish seeds in treatments with different stocking densities. Note: Numbers followed by the different superscript letters in the graph show significant difference (*P* < 0.05).

**Figure 3:**
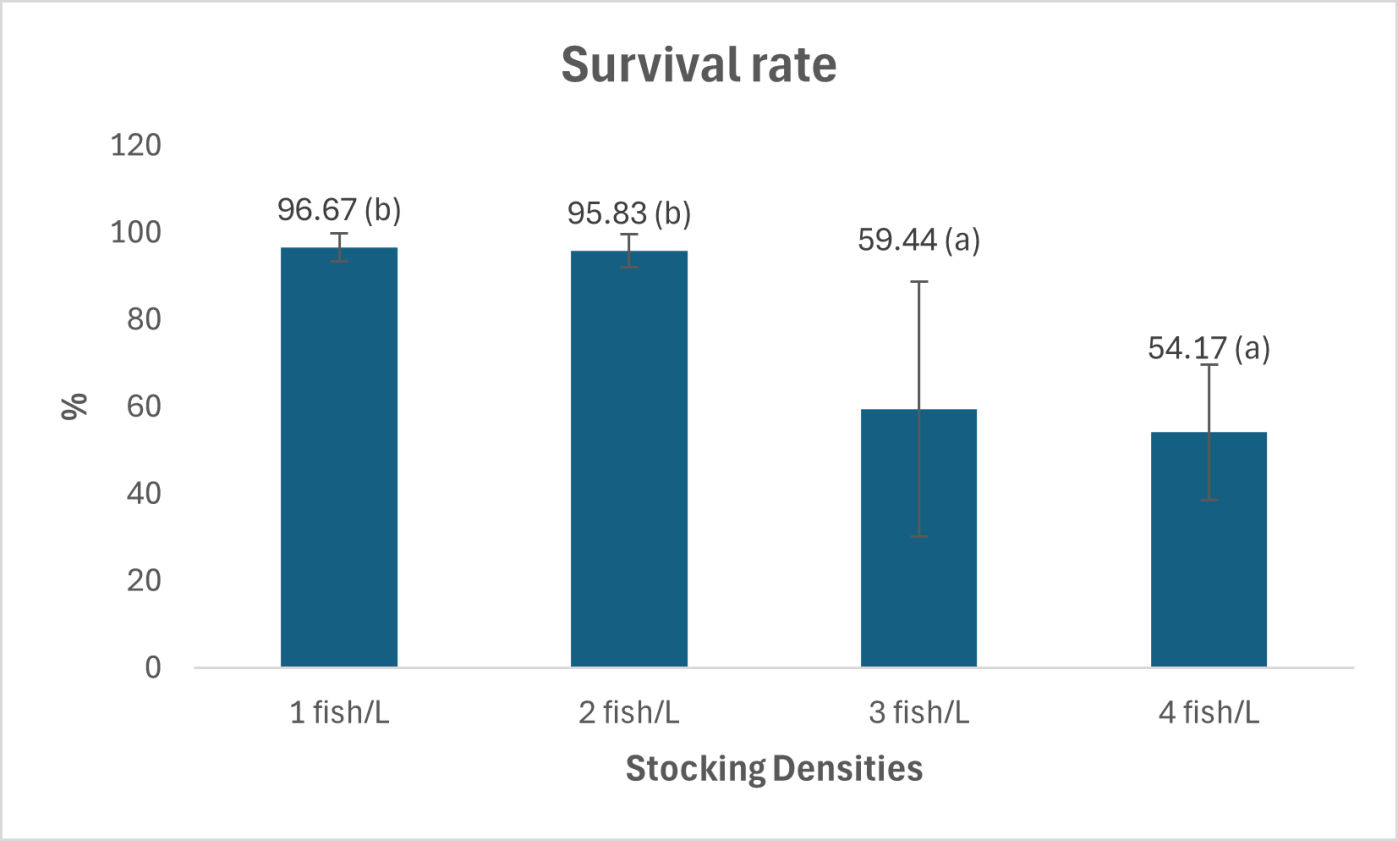
Survival of *Tor soro* fish kept at different stocking densities. Note: Numbers followed by the different superscript letters in the graph show significant difference (*P* < 0.05).

Figures 1, 2 and 3 provide an overview of the effect of density on fish growth. The highest growth rate and daily growth rate occurred at a density of one fish/L (2.2 ± 0.38 g and 1.17 ± 0.09%, respectively) and were significantly different from densities of three and four fish/L. Figures 1 and 2 also show that the growth rate tends to increase along with decreasing stocking density. The stocking density significantly affects the survival rate of *Tor soro* fry (*p* < 0.05). Figure 3 shows that the survival rates at stocking densities of three and four fish/L (54.17 ± 15.55% and 59.44 ± 29.28%, respectively) are lower and significantly different compared to those at densities of one and two fish/L (96.67 ± 3.19% and 95.83 ± 3.85%, respectively). Table 2 summarizes the effects of varying stocking densities on gross energy content and energy retention in Mahseer (*Tor soro*). The highest gross energy value (2,151 kcal/g) was observed at the lowest stocking density (one fish/L), representing a statistically significant increase (*p* < 0.05) compared to treatments with higher densities (two, three, and four fish/L).

Table 3 presents the proximate composition (water, ash, fat, and protein content) of *Tor soro* body tissue before and after exposure to varying stocking densities. At the start of the experiment, the whole-body water content was 72.47 ± 0.40%. By the end of the trial, water content exhibited an inverse relationship with stocking density: the lowest value (69.22 ± 0.53%) was recorded at one fish/L, while the highest (73.74 ± 0.21%) occurred at 4 fish/L (*p* < 0.05). Conversely, ash content increased with higher stocking densities. Initial ash levels (2.51 ± 0.07%) rose significantly, peaking at 4 fish/L (4.59 ± 0.16%), which was statistically distinct from all lower-density treatments (*p* < 0.05). The initial fat content of *Tor soro* was 8.79 ± 0.42%. By the end of the trial, fat content demonstrated a density-dependent decline, with significantly higher values in the one fish/L (9.48 ± 0.07%), two fish/L (9.09 ± 0.18%), and three fish/L (9.26 ± 0.23%) groups compared to the four fish/L group (6.63 ± 0.27%; *p* < 0.05). Concurrently, protein content shifted from an initial value of 13.64 ± 0.16% to a contrasting pattern: the highest post-treatment protein level (14.46 ± 0.05%) was observed in the three fish/L group. At the same time, the lowest occurred in the four fish/L group. These results indicate opposing lipid and protein retention trends under varying stocking densities.

**Table 2:** Gross energy of mahseer fish seeds reared at different stocking densities.

Stocking Density (fish/L)	Gross Energy (kcal/g)
1 (15 fish)	2,151 ± 1.50^d^
2 (30 fish)	1,958 ± 1.71^c^
3 (45 fish)	1,442 ± 2.83^a^
4 (60 fish)	1,680 ± 2.06^b^

**Notes.**

Numbers followed by the same superscript letters in the same column showed no significant difference (Duncan’s test *P* > 0.05).

**Table 3:** Proximate analysis of the body of *Tor soro* fry reared at different stocking densities.

Treatment	Tor soro Fish Body Proximate				
	Water content (%)	Ash (%)	Fat (%)	Proteins (%)	Carbohydrate (%)
Beginning	72.47 ± 0.40^c^	2.51 ± 0.07^a^	8.79 ± 0.42^b^	13.64 ± 0.16^a^	2.60 ± 0.71^b^
1 (15 fish)	69.22 ± 0.53^a^	3.24 ± 0.10^c^	9.48 ± 0.07^c^	15.19 ± 0.05^c^	2.86 ± 0.64^b^
2 (30 fish)	70.25 ± 0.14^b^	2.98 ± 0.11^b^	9.09 ± 0.18^bc^	15.37 ± 0.37^c^	2.32 ± 0.45^b^
3 (45 fish)	70.41 ± 0.33^b^	3.19 ± 0.26^bc^	9.26 ± 0.23^c^	16.46 ± 0.07^d^	0.69 ± 0.32^a^
4 (60 fish)	73.74 ± 0.21^d^	4.59 ± 0.16^d^	6.63 ± 0.27^a^	14.46 ± 0.05^b^	0.57 ± 0.26^a^

**Notes.**

Numbers followed by the same superscript letters in the same column showed no significant difference (Duncan’s test *P* > 0.05).

Initial superoxide dismutase (SOD) levels in *Tor soro* were measured at 2.56 ± 0.49 ng/mg (Table 4). By the experiment’s conclusion, SOD activity had increased differentially across treatment groups. Fish stocked at three fish/L (7.36 ± 0.40 ng/mg) and four fish/L (7.79 ± 0.51 ng/mg) exhibited the highest SOD levels, while groups at 1 fish/L (3.50 ± 0.89 ng/mg) and two fish/L (4.96 ± 0.48 ng/mg) showed comparatively lower increases. Similarly, baseline cortisol concentrations (5.56 ± 0.14 ng/ml) rose significantly by the end of the trial, with the highest stress responses observed in three fish/L and four fish/L treatments (9.05 ± 0.29 ng/ml and 9.20 ± 0.27 ng/ml, respectively). These cortisol levels were markedly elevated compared to one fish/L and two fish/L treatments (6.06 ± 0.10 ng/ml and 6.79 ± 0.13 ng/ml, respectively; *p* < 0.05). The divergent trends in antioxidant defense (SOD) and stress hormone (cortisol) responses suggest a density-dependent physiological trade-off under higher stocking intensities.

**Table 4:** Cortisol and SOD content in the body of *Tor soro* fry reared at different stocking densities.

Parameter	Treatment				
	Initial	1 fish/L	2 fish/L	3 fish/L	4 fish/L
Cortisol (ng/ml)	5.56 ± 0.14^a^	6.06 ± 0.19^b^	6.79 ± 0.13^c^	9.05 ± 0.29^d^	9.20 ± 0.27^d^
SOD (ng/mg)	2.56 ± 0.49^a^	3.50 ± 0.89^a^	4.96 ± 0.48^b^	7.36 ± 0.40^c^	7.79 ± 0.51^c^

**Notes.**

Numbers followed by the same superscript letters in in the same row showed no significant difference (*P* > 0.05).

Water quality parameters in *Tor soro* rearing tanks over the 60-day experimental period are summarized in Table 5. Water quality data for all parameters showed no significant differences between treatments (*P* < 0.05). Temperature remained stable across all stocking densities, ranging from 24.2 °C to 27.12 °C, within the optimal range for aquaculture. Dissolved oxygen (DO) levels were consistently maintained at 7.70–8.10 mg/L, ensuring adequate oxygenation for fish metabolism. Turbidity showed a positive correlation with stocking density, with the highest values observed in the three fish/L (5.14 ± 0.08 NTU) and four fish/L (5.12 ± 0.05 NTU) treatments, while the 1 fish/L group exhibited the lowest turbidity (5.06 ± 0.04 NTU). pH levels remained relatively stable, although the four fish/L treatment displayed the widest range (7.69 ± 0.52) compared to the one fish/L group (7.63 ± 0.48). Nitrite concentrations were narrowly distributed across treatments, average 0.104 ± 0.062 mg/L (one fish/L) to 0.112 ± 0.057 mg/L (four fish/L). Nitrate levels varied slightly, with the three fish/L treatment showing the highest concentrations (4.829 ± 0.427 mg/L) and the one fish/L group the lowest (4.302 ± 0.077 mg/L). Alkalinity ranged from 64.02 to 79.44 mg/L CaCO_3_, peaking in the two fish/L and three fish/L treatments, likely due to heightened biological activity. Total hardness was relatively uniform, with the two fish/L group exhibiting the highest values (125.6 ± 4.9 mg/L CaCO_3_) and the one fish/L group the lowest (110.9 ± 0.6 mg/L CaCO_3_). Total ammonia nitrogen (TAN) remained low and stable across all densities, fluctuating between 0.043 and 0.054 mg/L, indicating effective waste management. These findings suggest that water quality parameters were largely maintained within acceptable limits for *Tor soro* rearing, despite minor variations linked to stocking density.

**Table 5:** Water quality in *Tor soro* fry rearing tanks over a 60-day treatment at different stocking densities.

Parameters		Water quality		
	1 fish/L	2 fish/L	3 fish/L	4 fish/L
Temperature (°C)	25.67 ± 1.46^a^	25.72 ± 1.46^a^	26.22 ± 1.67^a^	25.76 ± 1.42^a^
Dissolved Oxygen (mg/L)	7.92 ± 0.09^a^	7.94 ± 0.18^a^	7.94 ± 0.21^a^	7.95 ± 0.20^a^
Turbidity (NTU)	5.06 ± 0.04^a^	5.07 ± 0.06^a^	5.14 ± 0.08^a^	5.12 ± 0.05^a^
pH	7.63 ± 0.48^a^	7.71 ± 0.56^a^	7.70 ± 0.58^a^	7.69 ± 0.52^a^
Nitrite (mg/L)	0.104 ± 0.062^a^	0.109 ± 0.054^a^	0.104 ± 0.058^a^	0.112 ± 0.057^a^
Nitrate (mg/L)	4.302 ± 0.077^a^	4.674 ± 0.292^a^	4.829 ± 0.427^a^	4.513 ± 0.276^a^
Alkalinity (mg/L CaCO_3_)	71.67 ± 7.65^a^	72.23 ± 7.19^a^	72.23 ± 7.21^a^	70.62 ± 6.60^a^
Total Hardness (mg/L CaCO_3_)	110.9 ± 0.6^a^	125.6 ± 4.9^a^	120.9 ± 0.1^a^	120.65 ± 10.3^a^
TAN (mg/L)	0.050 ± 0.005^a^	0.049 ± 0.004^a^	0.048 ± 0.004^a^	0.046 ± 0.003^a^

## Discussion

Stocking density critically influences fish growth and survival by modulating key factors such as spatial availability, feed access, stress levels, and water quality in aquaculture systems (Chowdhury, Roy & Chowdhury, 2020; Liu et al., 2019; Zahedi et al., 2019). Elevated stocking densities often correlate with reduced growth rates and, in extreme cases, heightened mortality due to intensified competition for food, oxygen, and space, compounded by deteriorating water quality. This competitive stress triggers physiological responses as fish expend energy to maintain homeostasis under suboptimal conditions, ultimately compromising growth performance. In this study, *Tor soro* reared at the highest density (four fish/L) exhibited significantly lower growth metrics (*p* < 0.05), including daily growth rate, final length, final weight, absolute length growth, and absolute weight growth. Dominance hierarchies likely exacerbated resource inequality, with dominant individuals monopolizing feed, leaving subordinates undernourished and stunted. Conversely, fish maintained at one fish/L and two fish/L demonstrated superior growth performance across all measured parameters (*p* < 0.05), reflecting minimal competition and optimal rearing conditions for normal development.

These findings align with broader evidence showing density-dependent growth patterns in aquaculture. For instance, goldfish (*Carassius auratus*) reared under varying stocking densities similarly exhibit divergent growth trajectories, underscoring the universal impact of population pressure on fish physiology. To optimize productivity and welfare, balancing stocking density with resource availability is essential for minimizing stress and maximizing yield in *Tor soro* and other cultured species. Survival rates serve as critical indicators of fish tolerance and adaptability to captive rearing conditions. In this study, lower stocking densities (one to two fish/L) yielded significantly higher survival rates compared to treatments with three and four fish/L (Table 1). The decline in survival at higher densities likely stems from elevated stress caused by spatial confinement, aggressive competition for food and space, and handling disturbances during sampling. Subordinate individuals unable to secure adequate nutrition may experience stunted growth and heightened susceptibility to mortality. Feed conversion ratio (FCR)—a metric reflecting feed utilization efficiency—worsened with increasing stocking density. Higher densities correlated with elevated FCR values, peaking at 2.95  ± 0.13 for 4 fish/L, whereas the lowest density (1 fish/L) achieved the most efficient FCR of 1.76 ± 0.13 (Table 1). These findings align with El-Dahhar et al. (2021), who reported similar trends in *Argyrosomus regius* (Asso, 1801) fingerlings, where increased stocking density reduced feed efficiency and heightened FCR values. Collectively, these results underscore the trade-off between rearing intensity and physiological performance, emphasizing the need to balance productivity with fish welfare in aquaculture systems.

*Tor soro* fry exhibited the highest gross energy content at the lowest stocking density (one fish/L: 2,151 kcal/g), with values declining progressively at higher densities (two to four fish/L; Haser et al., 2024). This trend underscores the significant impact of stocking density on energy dynamics, as increased population pressure necessitates greater energy expenditure for competition over food and space, leaving some individuals unable to secure adequate nutrition (Xue et al., 2021). Gross energy fluctuations reflect the interplay between dietary intake and energy allocation for growth, metabolic activity, and physiological maintenance. Fish receiving sufficient high-energy feed typically achieve rapid growth and optimal performance, while those with insufficient energy intake face compromised growth rates, diminished health, and reduced efficiency due to metabolic limitations. These findings highlight the critical balance between rearing intensity and energy utilization, emphasizing the need to optimize stocking densities to maximize growth potential and welfare in *Tor soro* aquaculture systems.

Proximate body composition analysis quantifies critical components such as water, ash, fat, and protein in fish tissue. Assessing the proximate composition of *Tor soro* after rearing at varying stocking densities provides insights into their nutritional status and physiological responses to environmental stressors. Results revealed that fish in the four fish/L treatment group demonstrated the highest water (73.74 ± 0.21%) and ash content (4.59 ± 0.16%) but the lowest fat (6.63 ± 0.27%) and protein (14.46 ± 0.05%) levels compared to lower-density treatments. This pattern suggests that muscle lipids may be partially replaced by water, reducing lean tissue mass—a phenomenon linked to energy reallocation under stress (Aliabad et al., 2022). These findings align with Chen et al. (2020), who observed similar shifts in tilapia (*Oreochromis niloticus*) under food scarcity, where elevated water and ash content coincided with depleted fat reserves. The reduced fat and protein content at high stocking density (four fish/L) implies energy deficiency, likely driven by inadequate feed access and heightened metabolic demands. Under such conditions, fish may mobilize fat and protein reserves to meet elevated energy requirements, compromising somatic growth and physiological resilience (Singh, Kesharwani & Keservani, 2017). Native to clear, rocky, fast-flowing waters, *Tor soro* is highly sensitive to environmental disturbances (Desrita Tamba et al., 2019), underscoring the importance of maintaining optimal water quality in captivity to mitigate stress and support health.

High stocking densities elevate stress biomarkers such as SOD and cortisol in fish (Yang et al., 2020). In this study, the significantly higher SOD and cortisol levels observed in the three and four fish/L treatment groups indicate heightened physiological stress in Torsoro. SOD, a critical antioxidant enzyme, catalyzes the dismutation of toxic superoxide radicals into less reactive species (*e.g.*, hydrogen peroxide and molecular oxygen), protecting cells from oxidative damage (Fujii, Homma & Osaki, 2022; Zheng et al., 2023). Elevated SOD activity under high-density conditions reflects an intensified oxidative stress response, as corroborated by studies on *Labeo rohita*, which similarly demonstrated increased SOD levels under crowded rearing conditions (Swain et al., 2022). Cortisol, a primary glucocorticoid stress hormone, serves as a key mediator of stress responses in fish. Its secretion rises under adverse conditions, triggering metabolic shifts that prioritize energy mobilization overgrowth (Hoseini et al., 2020). In this experiment, escalating stocking densities correlated with elevated cortisol production, likely disrupting body composition and impairing growth performance. Cortisol stimulates glycogenolysis in the liver and muscles, increasing blood glucose levels—a widely used proxy for stress assessment in aquaculture. This aligns with findings from Swain et al. (2022), who reported cortisol elevation in *Labeo rohita* under high stocking densities. The observed stress-induced energy reallocation (from somatic growth to survival mechanisms) underscores the trade-off between rearing intensity and physiological resilience, emphasizing the need to optimize stocking strategies to mitigate stress and sustain productivity. Water quality parameters during the study showed minimal variation across treatments (Table 5), indicating stable rearing conditions conducive to *Tor soro* cultivation. Established strategies, such as recirculating aquaculture systems (RAS), further enhance water quality control by minimizing waste accumulation and ensuring consistent oxygenation—critical for sustaining sensitive species under intensive stocking regimes.

High stocking density is a key factor influencing water quality in aquaculture systems. Increased biomass leads to higher metabolic waste production, oxygen demand, and organic matter accumulation, raising the risk of environmental deterioration. Haser et al. (2022) reported that dissolved oxygen decreased and ammonia increased in tanks with higher stocking densities of *Tor soro* fingerlings. Similarly, Indra et al. (2024) observed that elevated densities intensified competition for space and resources, impairing water quality through overcrowding and greater biological waste. Gharti et al. (2023) also found that pH and turbidity worsened under high-density conditions, highlighting the necessity of effective management strategies. Physiological responses further illustrate density-related stress. This corresponds with Barlaya et al. (2021), who found that lower densities promoted stable water quality and improved growth. These studies emphasize the interplay between environmental conditions and fish health under varying densities.

Continuous monitoring and responsive management are essential to mitigate these challenges. Even advanced systems such as recirculating aquaculture face difficulties maintaining optimal water quality at high biomass loads (Wang et al., 2019). Monitoring enables timely adjustments in aeration, feeding, waste removal, and water exchange to prevent parameters from reaching harmful levels (Atique, Lindholm-Lehto & Pirhonen, 2022; Mugwanya et al., 2022).

In the present study, water quality was successfully maintained within safe limits across all density treatments. Controlled feeding, ensuring feed was consumed within 10–15 min, daily siphoning of feces, continuous aeration, and routine 30% water exchange effectively minimized organic load, prevented ammonia buildup, and stabilized dissolved oxygen, ammonia, nitrite, and pH. These results demonstrate that while high stocking density increases environmental pressure, its negative effects can be alleviated through rigorous monitoring and consistent management. This aligns with existing literature, confirming that proactive husbandry practices are critical for sustaining optimal conditions in intensive aquaculture systems.

## Conclusions

This study demonstrates that stocking density critically influences growth performance, physiological stress responses, and overall health of *Tor soro* in aquaculture systems. Stocking densities of one fish/L and two fish/L optimize growth rates and survival while minimizing stress, as evidenced by improved feed conversion ratios (FCR) and stable physiological markers, including cortisol (6.06–6.79 ng/ml) and superoxide dismutase (SOD) activity (3.50–4.96 ng/mg). These conditions support efficient energy allocation toward growth rather than stress mitigation. In contrast, higher densities (three to four fish/L) exacerbate competition for food and space, degrade water quality, and trigger significant elevations in cortisol (9.05–9.20 ng/ml) and SOD (7.36–7.79 ng/mg), reflecting heightened oxidative and metabolic stress. The resultant energy diversion from somatic growth to survival mechanisms compromises growth performance (*e.g.*, reduced final weight and length) and increases mortality risk. These findings underscore the necessity of balancing stocking density with resource availability to sustain productivity and fish welfare in *Tor soro* aquaculture. Maintaining optimal densities ensures stable water quality, reduces physiological strain, and enhances feed efficiency, aligning with broader principles of sustainable aquaculture management.

##  Supplemental Information

10.7717/peerj.21034/supp-1 Supplemental Information 1 Raw Data

10.7717/peerj.21034/supp-2 Supplemental Information 2 ARRIVE checklist

## Additional Information and Declarations

### Data Availability

The following information was supplied regarding data availability: The raw measurements are available in the Supplemental File.

## References

[ref-1] Abass Z, Shah TH, Bhat FA, Ramteke K, Magloo AH, Hamid I, Wanjari RN, Somasundharam I (2024). The mahseer: the tiger of water-an angler’s delight in the Himalayas and the undisputed king of sport fishing. Fisheries Research.

[ref-2] Abd El-Hack ME, El-Saadony MT, Nader MM, Salem HM, El-Tahan AM, Soliman SM, Khafaga AF (2022). Effect of environmental factors on growth performance of Nile tilapia (*Oreochromis niloticus*). International Journal of Biometeorology.

[ref-3] Akmal Y, Muliari M, Humairani R, Zulfahmi I, Burhanuddin AI, Budimawan B, Batubara AS (2022). Species authentication of *Tor spp*, (family Cyprinidae) in Indonesia based on osteocranium structure and biometric data. Zoologischer Anzeiger.

[ref-4] Aliabad HS, Naji A, Mortezaei SRS, Sourinejad I, Akbarzadeh A (2022). Effects of restricted feeding levels and stocking densities on water quality, growth performance, body composition and mucosal innate immunity of Nile tilapia (*Oreochromis niloticus*) fry in a biofloc system. Aquaculture.

[ref-5] Arifin ZA, Mumpuni FS, Sofian A, Cahyanti W, Soebakti Hasan OD (2020). Embryonic development of torsoro fish (*Tor soro*) at different incubation temperatures. Media Akuakultur.

[ref-6] Arnenda GL, Kurniawan K, Radona D, Cahyanti W, Prakoso VA, Ath-Thar MH, Putri FP, Prihadi TH, Kusmini II (2023). Growth performance of hatchery-reared mahseer (*Tor soro*) based on different cultural periods. E3s Web of Conferences.

[ref-7] Atique F, Lindholm-Lehto P, Pirhonen J (2022). Is aquaponics beneficial in terms of fish and plant growth and water quality in comparison to separate recirculating aquaculture and hydroponic systems?. Water.

[ref-8] Barlaya G, Narasimhan S, Basumatary P, Huchchappa R, Kumar A, Kannur H (2021). Effect of stocking density on the growth and survival of the critically endangered peninsular *carphypselobarbus pulchellus* (day, 1870) in fingerling rearing. Aquaculture Research.

[ref-9] Chasanah E, Fithriani D, Poernomo A, Jeinie MH, Huda N (2021). The nutritional profile of Indonesian salmon vanJava mahseer T. Soro species. Slovak Journal of Food Sciences/Potravinarstvo.

[ref-10] Chen JX, Feng JY, Zhu J, Luo L, Lin SM, Wang DS, Chen YJ (2020). Starch to protein ratios in practical diets for genetically improved farmed Nile tilapia *Oreochromis niloticus*: effects on growth, body composition, peripheral glucose metabolism and glucose tolerance. Aquaculture.

[ref-11] Chowdhury MA, Roy NC, Chowdhury A (2020). Growth, yield and economic returns of striped catfish (*Pangasianodon hypophthalmus*) at different stocking densities under floodplain cage culture system. Egyptian Journal of Aquatic Research.

[ref-12] Desrita Tamba IS, Muhtadi A, Ariyanti J, Leidonald R (2019). Diversity and habitat condition of Tor Fish (Tor spp.) in the upstream of Wampu Waters, North Sumatra, Indonesia. IOP Conference Series: Earth and Environmental Science.

[ref-13] Diao W, Jia R, Hou Y, Dong Y, Li B, Zhu J (2023). Effects of stocking density on the growth performance, physiological parameters, antioxidant status and lipid metabolism of *Pelteobagrus fulvidraco* in the integrated rice-fish farming system. Animals.

[ref-14] Dwivedi A (2021). Morphometric assessment of golden mahseer populations in the Ganga River Basin, India. Fisheries.

[ref-15] El-Dahhar AA, El-Ebiary ESH, Abdel-Rahim MM, Refaee WM, Lotfy AM (2021). Stocking density effects on survival, growth performance, feed utilization, and carcass composition of meagre, *Argyrosomus regius* (Asso, 1801) fingerlings reared in fiberglass tanks using underground saltwater. Aquaculture, Aquarium, Conservation & Legislation.

[ref-16] El Nouman BA, Egbal OA, Sana YA, Anwar MS, Eman AA, Yosif FA (2021). Determine the optimal density of Nile tilapia (*Oreochromis niloticus*) fingerlings cultured in floating cages. Natural Resources.

[ref-17] Everard M, Pinder A, Claussen J, Orr S (2021). Assessing the societal benefits of mahseer (Tor spp.) fishes to strengthen the basis for their conservation. Aquatic Conservation Marine and Freshwater Ecosystems.

[ref-18] Fadir RM, Haser TF, Febri SP, Prihadi TH, Cahyanti W (2022). Dinamic of water quality on maintenance jurung fish (Tor soro) that maintained on various recirculation systems. Acta Aquatica: Aquatic Sciences Journal.

[ref-19] Fujii J, Homma T, Osaki T (2022). Superoxide radicals in the execution of cell death. Antioxidants.

[ref-20] Gharti K, Yan L, Li K, Boonpeng N, Liu L (2023). Growth and muscle quality of grass carp (*Ctenopharyngodon idella*) in in-pond raceway aquaculture and traditional pond culture. Water.

[ref-21] Groot R, Lyons P, Schrama JW (2021). Digestible energy *versus* net energy approaches in feed evaluation for rainbow trout (*Oncorhynchus mykiss*). Animal Feed Science and Technology.

[ref-22] Gullian-Klanian MAAC, Arámburu-Adame C (2013). Performance of Nile tilapia *Oreochromis niloticus* fingerlings in a hyper-intensive recirculating aquaculture system with low water exchange. Latin American Journal of Aquatic Research.

[ref-23] Haser TF, Supriyono E, Nirmala K, Prihadi TH, Budiardi T, Azmi F, Nurdin MS (2022). Effects of different stocking densities on growth performance of tor soro fingerlings under recirculation aquaculture system. IOP Conference Series Earth and Environmental Science.

[ref-24] Haser TF, Supriyono E, Nirmala K, Prihadi TH, Budiardi T, Syamsudin R, Nurdin MS (2024). Enhancement of body performance and growth performance of juvenile mahseer (*Tor soro*) using differently colored containers. Fisheries and Aquatic Sciences.

[ref-25] Hoseini SM, Mirghaed AT, Ghelichpour M, Pagheh E, Iri Y, Kor A (2020). Effects of dietary tryptophan supplementation and stocking density on growth performance and stress responses in rainbow trout (*Oncorhynchus mykiss*). Aquaculture.

[ref-26] Indra A, Dewi N, Mahasri G, Rahardja B, Arifin O (2024). The effect of stocking densities on growth and survival rate of thai mahseer (*Tor tambroides*) during nursery stage. IOP Conference Series Earth and Environmental Science.

[ref-27] Jaafar F, Na-Nakorn U, Srisapoome P, Amornsakun T, Duong TY, Gonzales-Plasus MM, Hoang DH, Parhar IS (2021). A current update on the distribution, morphological features, and genetic identity of the Southeast Asian mahseers, Tor species. Biology.

[ref-28] Jia R, Wang L, Hou Y, Feng W, Li B, Zhu J (2022). Effects of stocking density on the growth performance, physiological parameters, redox status and lipid metabolism of *Micropterus salmoides* in integrated rice–fish farming systems. Antioxidants.

[ref-29] Liu Y, Liu H, Wu W, Yin J, Mou Z, Hao F (2019). Effects of stocking density on growth performance and metabolism of juvenile Lenok (*Brachymystax lenok*). Aquaculture.

[ref-30] Majhi SS, Singh SK, Biswas P, Debbarma R, Parhi J, Ngasotter S, Waikhom G, Meena DK, Devi AG, Mahanand S, Xavier KAM, Patel AB (2023). Effect of stocking density on growth, water quality changes and cost efficiency of butter catfish (*Ompok bimaculatus*) during seed rearing in a biofloc system. Fishes.

[ref-31] Misra HP, Fridovich I (1972). The role of superoxide anion in the autoxidation of epinephrine and a simple assay for superoxide dismutase. Journal of Biological chemistry.

[ref-32] Muchlisin ZA, Nur FM, Maulida S, Handayani LS, Rahayu SR (2022). Mahseer, the history of the king of the river. E3S Web of Conferences. Vol. 339. The 2nd International and National Symposium on Aquatic Environment and Fisheries (INSAEF), 27–28 September 2021.

[ref-33] Mugwanya M, Dawood M, Kimera F, Sewilam H (2022). A review on recirculating aquaculture system: influence of stocking density on fish and crustacean behavior, growth performance, and immunity. Annals of Animal Science.

[ref-34] Rai A, Jha B, Shrestha K, Lagunes-Díaz E (2025). Identifying early life habitat of golden mahseer fish Tor putitora (Hamilton, 1822) in South Asia: implications for conservation. Ecology and Evolution.

[ref-35] Riar MGS, Raushon NA, Paul SK (2021). Effect of stocking density on growth performance and the survival of golden mahseer, *Tor putitora* (Hamilton) fry. Asian Journal of Fisheries and Aquatic Research.

[ref-36] Setijaningsih L, Adiyana K, Thesiana L, Ardi I, Puspaningsih D, Setiadi E, Taufik I, Yamin M, Kurniasih T (2023). Study of bottom substrate variation in zero water discharge aquaculture for mahseer fish *Tor soro* nursery. Polish Journal of Environmental Studies.

[ref-37] Singh P, Kesharwani RK, Keservani RK (2017). Protein, carbohydrates, and fats: energy metabolism. Sustained energy for enhanced human functions and activity.

[ref-38] Subagja J, Imanudin EM, Kurniawan K, Soeprijanto A, Maemunah Y (2021). Determining the optimum temperature for growth, feed efficiency and survival of domesticated Indonesian mahseer, *Tor soro* larvae. Indonesian Aquaculture Journal.

[ref-39] Sundh H, Finne-Fridell F, Ellis T, Taranger GL, Niklasson L, Pettersen EF, Wergeland HI, Sundell K (2019). Reduced water quality associated with higher stocking density disturbs the intestinal barrier functions of Atlantic salmon (*Salmo salar* L.). Aquaculture.

[ref-40] Swain HS, Das BK, Upadhyay A, Ramteke MH, Kumar V, Meena DK, Sarkar UK, Chnada NK, Rawat KD (2022). Stocking density mediated stress modulates growth attributes in cage reared *Labeo rohita* (Hamilton) using multifarious biomarker approach. Scientific Reports.

[ref-41] Tan C, Sun D, Tan H, Liu W, Luo G, Wei X (2018). Effects of stocking density on growth, body composition, digestive enzyme levels and blood biochemical parameters of *Anguilla marmorata* in a recirculating aquaculture system. Turkish Journal of Fisheries and Aquatic Sciences.

[ref-42] Wang Y, Xu G, Nie Z, Shao N, Li Q, Xu P (2019). Growth performance of bluntnose black bream, channel catfish, yellow catfish, and largemouth bass reared in the in-pond raceway recirculating culture system. North American Journal of Aquaculture.

[ref-43] Xue S, Ding J, Li J, Jiang Z, Fang J, Zhao F, Mao Y (2021). Effects of life, artificial and mixed feeds on the growth and energy budget of *Penaeus vannamei*. Aquaculture Reports.

[ref-44] Yang Q, Guo L, Liu BS, Guo HY, Zhu KC, Zhang N, Jiang SG, Zhang DC (2020). Effects of stocking density on the growth performance, serum biochemistry, muscle composition and HSP70 gene expression of juvenile golden pompano *Trachinotus ovatus* (Linnaeus, 1758). Aquaculture.

[ref-45] Zahedi S, Akbarzadeh A, Mehrzad J, Noori A, Harsij M (2019). Effect of stocking density on growth performance, plasma biochemistry and muscle gene expression in rainbow trout (*Oncorhynchus mykiss*). Aquaculture.

[ref-46] Zheng M, Liu Y, Zhang G, Yang Z, Xu W, Chen Q (2023). The applications and mechanisms of superoxide dismutase in medicine, food, and cosmetics. Antioxidants.

